# Tools to improve the diagnosis and management of T-cell mediated adverse drug reactions

**DOI:** 10.3389/fmed.2022.923991

**Published:** 2022-10-13

**Authors:** Ana Maria Copaescu, Moshe Ben-Shoshan, Jason A. Trubiano

**Affiliations:** ^1^Department of Infectious Diseases, Centre for Antibiotic Allergy and Research, Austin Health, Heidelberg, VIC, Australia; ^2^Division of Allergy and Clinical Immunology, Department of Medicine, McGill University Health Centre (MUHC), Montreal, QC, Canada; ^3^The Research Institute of the McGill University Health Centre, McGill University Health Centre (MUHC), Montreal, QC, Canada; ^4^Division of Allergy, Immunology and Dermatology, Montreal Children’s Hospital, McGill University Health Centre (MUHC), Montreal, QC, Canada; ^5^Department of Oncology, Sir Peter MacCallum Cancer Centre, The University of Melbourne, Parkville, VIC, Australia; ^6^Department of Medicine, Austin Health, The University of Melbourne, Heidelberg, VIC, Australia; ^7^The National Centre for Infections in Cancer, Peter MacCallum Cancer Centre, Melbourne, VIC, Australia

**Keywords:** drug allergy, *in vivo*, *ex vivo*, diagnostic tools, delayed hypersensitivity reaction, drug reaction with eosinophilia and systemic symptoms (DRESS), Stevens-Johnson syndrome (SJS) and toxic epidermal necrolysis (TEN), severe cutaneous adverse reactions (SCARs)

## Abstract

Delayed drug T-cell immune-mediated hypersensitivity reactions have a large clinical heterogeneity varying from mild maculopapular exanthema (MPE) to severe cutaneous adverse reactions (SCARs) such as acute generalized exanthematous pustulosis (AGEP), drug reaction with eosinophilia and systemic symptoms (DRESS) and severe skin necrosis and blistering as seen in Stevens-Johnson syndrome (SJS) and toxic epidermal necrolysis (TEN). Given the knowledge gaps related to the immunopathogenesis of these conditions, the absence of validated diagnostic tools and the significant associated morbidity and mortality, patients with SCARs often have limited drug choices. We performed a comprehensive review aiming to evaluate *in vivo* diagnostic tools such as delayed intradermal skin and patch testing and *ex vivo/in vitro* research assays such as the lymphocyte transformation test (LTT) and the enzyme-linked ImmunoSpot (ELISpot) assay. We searched through *PubMed* using the terms “drug allergy,” “*in vivo*” and “*ex vivo*” for original papers in the last 10 years. A detailed meticulous approach adapted to the various clinical phenotypes is recommended for the diagnostic and management of delayed drug hypersensitivity reactions. This review highlights the current diagnostic tools for the delayed drug hypersensitivity phenotypes.

## Introduction

Delayed immune-mediated drug hypersensitivity reactions (DHR) are inflammatory reactions with a predominant manifestation in the skin that can be associated with systemic manifestations, and are hypothesized to be T-cell mediated. These reactions are not anticipated and not dependent on the dose administered (1).

Severe cutaneous adverse reactions (SCARs) are DHR that cause severe damage to the skin and/or internal organs and are associated with significant acute and long-term morbidity and increased mortality risk (2). Risk factors include cystic fibrosis, severe asthma, chronic lymphatic leukemia, human immunodeficiency virus or genetic susceptibility (3). For the purpose of this review, we will focus on mild maculopapular exanthema (MPE) as well as SCAR syndromes: acute generalized exanthematous pustulosis (AGEP), drug reaction with eosinophilia and systemic symptoms (DRESS) and Stevens-Johnson syndrome (SJS) and toxic epidermal necrolysis (TEN). Our main goal is to portray the diagnostic methods, including a description of the currently used clinical skin testing and novel investigational *ex vivo* methods for the delayed DHR.

## Methods

We formulated a research question focusing on the available diagnostic tools aimed to improve the diagnosis and management of delayed T-cell mediated drug reactions. The objective of the comprehensive review was established using the PICO method, including population, interventions, comparators and outcomes. We searched *PubMed* for peer-reviewed original articles with the terms drug, antibiotic, antimicrobial, sulfonamide, non-steroidal anti-inflammatory, anti-epileptic or anti-convulsant; allergy, hypersensitivity or T-cell mediated; and *in vivo* as well as *ex vivo* diagnostic methods.

We used the key words: {[drug*(Title/Abstract)] OR [antibiotic*(Title/Abstract)] OR [antimicrobial*(Title/Abstract)] OR [sulfonamide*(Title/Abstract)] OR [non-steroidal anti-inflammator*(Title/Abstract)] OR [amoxicillin* (Title/Abstract)] OR [anti-epileptic*(Title/Abstract)] OR [anti-convulsant*(Title/Abstract)]} AND {[*ex vivo* (Title/Abstract)] OR [*in vitro* (Title/Abstract)] OR [skin testing*(Title/Abstract)] OR [patch testing*(Title/Abstract)] OR [enzyme-linked immunoSpot assay*(Title/Abstract)] OR [ELISpot(Title/Abstract)] OR [lymphocyte transformation test*(Title/Abstract)] OR [lymphocyte proliferation* (Title/Abstract)] OR [stimulation test*(Title/Abstract)] OR [IFN*(Title/Abstract)] OR [flow cytometry*(Title/Abstract)]} AND {[allergy*[Title/Abstract)] OR [hypersensitivity*(Title/Abstract)] OR [T-cell mediated*(Title/Abstract)]}.

**FIGURE 1:**
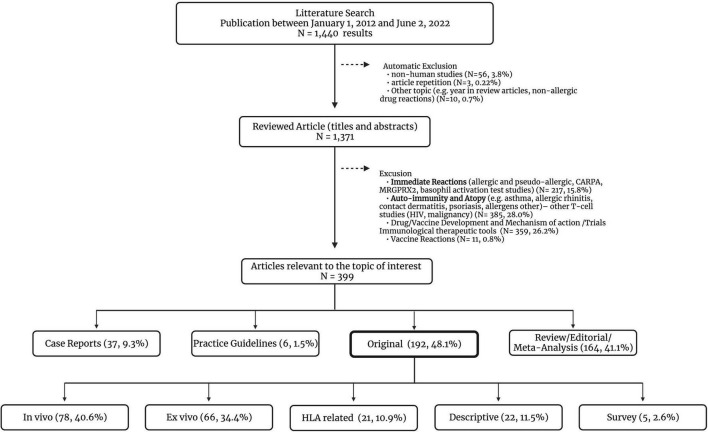
Comprehensive literature review–article selection. CARPA, complement activation-related pseudoallergy; HIV, human immunodeficiency virus; HLA, human leukocyte antigen; MRGPRX2, Mas-related G-protein coupled receptor member X2. The *ex vivo* original studies can also describe the use of *in vivo* diagnostic tools in the methods or study design.

Articles relevant to the topic of interest were examined following the inclusion criteria: (1) original human studies (pediatric and adult population), (2) academic articles published in peer-reviewed journals, (3) available in English or French language, and (4) published between January 1st 2012 and June 2nd 2022. The search provided 1,440 results (Figure 1). The first screening was based on the titles and abstracts followed by a second round of screening performed by reviewing the full-text articles for selected studies. For the purpose of this study, meta-analysis-based research articles were not considered in the original studies subcategory. Articles on immediate and vaccine hypersensitivity were excluded as these were considered beyond the scope of this review. To better illustrate the existing literature, original articles were further sub-categorized in studies containing information on *in vivo* tools, *ex vivo* tools and HLA-related research. The descriptive/epidemiological reports published that did not address any diagnostic tools were added to another subgroup (Figure 1).

## Delayed hypersensitivity reactions

**FIGURE 2:**
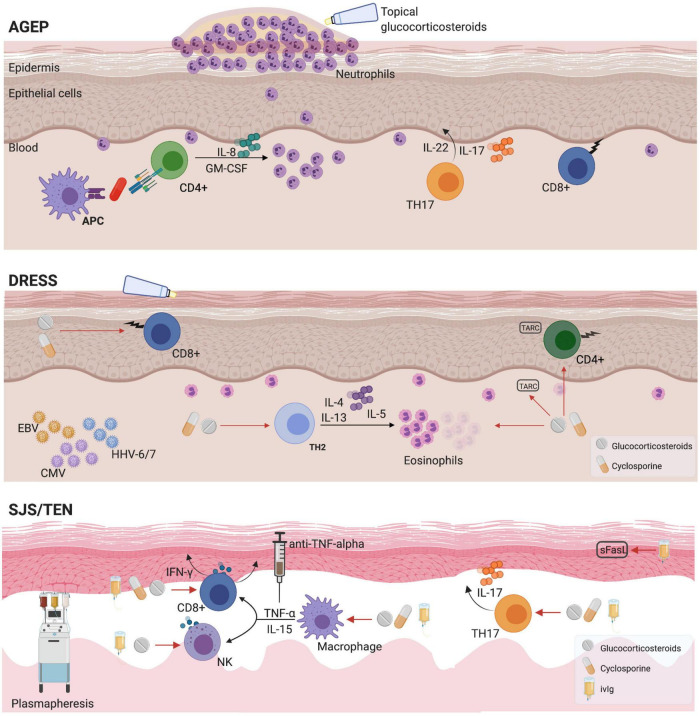
Mechanisms and pharmacological management for T-cell mediated reactions. AGEP, acute generalized exanthematous pustulosis; APC, antigen presenting cell; CMV, cytomegalovirus; DRESS, drug reaction with eosinophilia and systemic symptoms; EBV, Epstein-Barr virus; HHV, Human Herpesvirus; GM-CSF, granulocyte macrophage-colony stimulating factor; IFN-γ, Interferon gamma; IL, interleukin; sFasL, soluble Fas ligand; SJS, Stevens-Johnson syndrome; TARC, thymus and activation-regulated chemokine; TEN, toxic epidermal necrolysis; TNF, tumor necrosis factor.

Delayed hypersensitivity reactions can occur hours to days following exposure to a drug or drug metabolite. It is hypothesized that uncontrolled T-cell production triggers the different immune manifestations (Figure 2). Matured antigen-presenting cells such as dendritic cells and macrophages interact with antigen-specific T CD4+ helper cells as well as CD8+ cytotoxic T-cells leading to drug-specific cell-mediated immunity (4). While adaptive immunity plays an essential role, an implication of the innate immune response has been demonstrated *in vitro* for agents such as allopurinol (5).

There is a limited number of cohort studies that focus on providing a better understanding of the incidence, clinical description and mortality of DHR. The majority of the data is extrapolated from small older studies. Some reports suggest that drug-induced SCARs are less prevalent in the pediatric population compared to an adult population (6–9). A description of the main delayed drug related T-cell mediated hypersensitivity reactions is portrayed in Table 1 with an illustration of the immunopathogenesis and treatment options in Figure 2.

**TABLE 1:** Delayed drug related T-cell mediated hypersensitivity reactions.

Phenotype	Incidence	Clinical description	Average latency	Mortality	Skin biopsy	Common drugs (10)	Clinical score	Laboratory Investigations	*In vivo* Tools	*Ex vivo* tools	HLA association	Management options
MPE	2% (12)	Maculopapular erythematous eruption that can be associated with pruritus and/or mild eosinophilia	4–12 days	n/a	Vacuolar interface dermatitis and tissue eosinophilia	Antibiotics (penicillins, cephalosporins, sulfonamides); anticonvulsants	Naranjo score	CBC + Diff (Eosinophils)	IDT–Delayed reading PT Drug Challenge	n/a	n/a	Drug withdrawal Symptomatic treatment◆ Treating through
AGEP	1–5/million/ year (17)	Non-follicular sterile pustular rash over widespread erythema, fever and/or biological abnormalities	Hours–2 days (aminopenicillins) +2 weeks	2–4% (21, 22)	Spongiform subcorneal and/or intraepidermal pustules; perivascular and interstitial infiltrate	Antibiotics (penicillins, cephalosporins); antimycotics; other (diltiazem, oxicam, analgesics)	Naranjo score AGEP validation score	CBC + Diff (Neutrophils)	IDT–Delayed reading PT Drug Challenge♣	ELISpot LTT	n/a	Drug withdrawal Symptomatic treatment◆
DRESS	09-2/100,000 (32, 188)	Erythematous urticaria-like or violaceous skin eruption, facial and extremity edema, lymphadenopathy, fever, biological abnormalities and internal organ involvement.	2–8 weeks (<2 weeks antibiotics and contrast product)	3–10% (9, 189)	Interface dermatitis with basal vacuolization	Anticonvulsants; antibiotics (sulfonamides, vancomycin, minocycline); allopurinol	Naranjo score RegiSCAR score	CBC + Diff (Eosinophils) Liver panel Renal panel	IDT–Delayed reading PT Drug Challenge♣	ELISpot LTT	A*32:01 (84) (Vancomycin) B*58:01 (190) (Allopurinol) B*13:01 (191) (Dapsone) A*31:01 (Carbamazepine)	Drug withdrawal Symptomatic treatment◆ Systemic glucocorticoids (36) Cyclosporine (40)
SJS/TEN	2–7/million/ year (15)	Skin necrosis, skin detachment (Nikolsky sign) and blistering of the mucous membranes accompanied by serious systemic manifestations	4–28 days	30% (47)	Keratinocyte necrosis (partial to full-thickness necrosis of all epidermis layers)	Allopurinol; anticonvulsants; antibacterial sulfonamides; nevirapine; NSAIDs; antituberculosis agents	Naranjo score SCORTEN	CBC + Diff Liver panel Renal panel	PT	ELISpot LTT	B*15:02 (191) (Carbamazepine) B*58:01 (190) (Allopurinol)	Drug withdrawal Supportive wound care (51, 52) IVIG (54) Systemic glucocorticoids and IVIG (55) Cyclosporine (56, 57) TNF inhibitor (58)

AGEP, acute generalized exanthematous pustulosis; ALDEN, algorithm of drug causality for epidermal necrolysis; CBC, complete blood count; Diff, differential; DRESS, drug reaction with eosinophilia and systemic symptoms; ELISpot, enzyme-linked ImmunoSpot; IDT, intradermal testing, IVIG, intravenous immunoglobulin; LTT, lymphocyte transformation test; MPE, maculopapular exanthema; Naranjo score, the Adverse Drug Reaction (ADR) Probability Scale; PT, patch testing; RegiSCAR, European Registry of Severe Cutaneous Adverse Reactions; SCORTEN, Score of toxic epidermal necrosis; SJS, Stevens-Johnson syndrome; TEN, toxic epidermal necrolysis; TNF, tumor necrosis factor.

♣ In specific cases were investigations provide conclusive results, drug challenge can be considered with less likely or alternative drugs.

◆Symptomatic treatment consists of emollients, moderate to high-potency topical corticosteroid and second generation non-sedating oral antihistamines.

^1^Peter et al. (10); ^2^Bigby et al. (12); ^3^Sidoroff et al. (17); ^4^Saissi et al. (21); ^5^Sidoroff et al. (22); ^6^Wolfson et al. (32); ^7^Muller et al. (188); ^8^Kim et al. (9); ^9^Chiou et al. (189); ^10^Konvinse et al. (84); ^11^Hung et al. (204); ^12^Zhang et al. (205); ^13^Shiohara and Kano (36); ^14^Kuschel and Reedy (40); ^15^Rzany et al. (48); ^16^Sekula et al. (47); ^17^Schwartz et al. (51); ^18^Seminario-Vidal et al. (52); ^19^Huang et al. (54); ^20^Micheletti et al. (55); ^21^Gonzalez-Herrada et al. (56); ^22^Ng et al. (57); ^23^Wang et al. (58).

### Maculopapular exanthema

#### Clinical description

The MPE, morbilliform drug eruption or benign exanthem is the most common benign skin reaction associated with drugs. This condition is characterized by a maculopapular erythematous eruption that can become widespread and confluent and can be associated with pruritus and/or mild eosinophilia (10). The onset of the reaction typically occurs in the first 7–10 days of treatment for patients not previously exposed to the medication. However, in previously sensitized individual, re-exposure can lead to a skin eruption as rapid as 6–72 h after treatment initiation. In the pediatric population, viral exanthemas are an important differential diagnosis (11).

#### Epidemiology

Early studies suggest a prevalence of 2% for cutaneous drug eruptions in general (12), with up to 90% representing a mild phenotype. However, there is limited recent reliable data describing this non-severe type phenotypes. Another aspect is the non-immune mediated nature of some MPE that may result in overestimating the prevalence of this condition (13).

#### Drugs

All drug categories could, in theory, induce a skin eruption and there is a fine line between a recognized side effect and a mild skin hypersensitivity reaction. However, few studies that focus on a limited number of drugs have demonstrated how drugs induce T-cell mediated reaction mainly looking at antibiotics (penicillins, cephalosporins, sulfonamides) and anticonvulsants.

#### Management

Treating through in MPE is part of the accepted management options especially when the treatment alternatives could jeopardize the quality of the treatment or the treatment outcome (14, 15). The skin manifestation can be controlled with oral second-generation antihistamines as well as topical corticosteroids (15). A multidisciplinary approach is suggested for all delayed hypersensitivity conditions from MPE to TEN. Specialists implicated in the management vary depending on the organ involvement with allergy immunology, dermatology and infectious disease usually at the center of the management team (16).

### Acute generalized exanthematous pustulosis

#### Clinical description

The AGEP is a non-follicular, sterile, pustular rash over widespread erythema, with a preference for the flexural folds. This condition can be accompanied by systemic symptoms such as fever and/or biological abnormalities (10). A validation score from the EuroSCAR group criteria can be used to confirm the clinical diagnostic for AGEP cases (17). Part of the differential diagnosis of pustules localized on an erythematous skin is generalized pustular psoriasis (GPP), a rare subtype of psoriasis (18). During the initial clinical presentation, AGEP and GPP can be difficult to distinguish. The clinical evolution, with a shorter disease course for AGEP, as well as the biopsy with psoriasiform changes of the epidermis seen with GPP and absent in AGEP, allows the clinician to clarify the diagnosis (19, 20).

#### Epidemiology

A landmark study for AGEP comes from the 2001 EuroSCAR group that reports an incidence of 1–5 cases per million persons per year (17). The mortality rate was reported to be 2–4% (21, 22) while understanding that this condition has a favorable prognosis following culprit drug withdrawal (23).

#### Drugs

Multiple agents have been associated with AGEP (17) with antibiotics and antimycotics commonly described (21, 22). The short latency period for AGEP and certain specific clinical characteristics are considered agent specific (24). Case reports have described an association with infections (viral, bacterial or parasitic), spider insect bites and contrast agents (24).

#### Management

The main goal is to offer supportive care and to control the skin inflammation and pruritus. Similar to MPE, topical medium potency corticosteroids and second-generation antihistamines are commonly prescribed (25). In a retrospective review of electronic medical records from Singapore of 43 AGEP cases, where 9 (21%) patients were treated with systemic corticosteroids, the use of systemic corticosteroids compared with topical corticosteroids was associated with a reduction in the hospital stay (26). During the acute reaction, a skin biopsy can aid with the identification of the underlying phenotype. While this is not routinely performed for the mild drug eruption or for some of the classic manifestations, the histopathologic findings can support the diagnostic of a drug related reaction particularly in atypical cases or when GPP is suspected (Table 1).

### Drug reaction with eosinophilia and systemic symptoms

#### Clinical description

Drug reaction with eosinophilia and systemic symptoms or drug-induced hypersensitivity syndrome (DIHS) is a polymorphic erythematous urticaria-like or violaceous skin eruption that can progress to exfoliative dermatitis, facial and extremity edema. Patients can present with lymphadenopathy as well as fever, biological abnormalities and internal organ involvement. It is suggested that reactivation of viruses from the *Herpesviridae* family such as human herpesvirus (HHV)-6, HHV-7, Epstein-Barr virus (EBV), cytomegalovirus (CMV) play a major role in the pathogenesis (27, 28). This condition is characterized by a delayed onset, the time from the drug exposure varying from 2 to 6 weeks (29). Recent reports have described a shorter latency period (less than 15 days) for antibiotics and contrast agents (30). The RegiSCAR is calculated using clinical and laboratory data to estimate the probability of this condition (definite, probable, possible, or no case) (31).

#### Epidemiology

There are no large cohort studies or registries for DRESS. Using electronic health records, a recent report calculated the incidence at 2/100,000 (32) and a Spanish pharmacovigilance program described an incidence of 4/10,000 patients (33). The incidence of DRESS is drug and population dependent.

#### Drugs

The primary culprit drugs are antibiotics and anticonvulsants (32) as well as allopurinol (34). Recently, other agents such as contrast product have been described (35).

#### Management

Drug withdrawal is an essential part of acute management with patients often being restricted in terms of future drug options. The culprit agents and all possible cross-reactive drugs are avoided. As multiple organ involvement is frequent, systemic corticosteroids are usually initiated besides the usual supportive care (36–38). For refractory cases of DRESS with persistent elevated liver function, viral infections should be rule out as possible mimickers include infectious mononucleosis (EBV), CMV, and HIV (39). Case reports and small cases series demonstrate a role for cyclosporine as a second line agent (40, 41). There might be a role for other immunosuppressive agents but no randomized trials have showed a benefit and they are not part of routine management.

### Stevens-Johnson syndrome and toxic epidermal necrolysis

#### Clinical description

Stevens-Johnson syndrome and toxic epidermal necrolysis are characterized by skin necrosis, skin detachment (positive Nikolsky sign) and blistering of the mucous membranes accompanied by serious systemic manifestations. The mortality for this condition can reach 30–50% (42). The distinction between SJS and TEN is determined by affected body surface area (BSA): 1–10% for SJS, 10–30% for SJS/TEN overlap and >30% for TEN (10). The time interval from drug exposure to the development of symptoms can vary from 4 to 28 days and in a third of cases no causal agent is identified (29). In the pediatric population, *Mycoplasma pneumoniae* infection has been associated with SJS (43). A clinical score (SCORTEN) can be calculated to indicate prognostic value (44). The ALDEN score is an algorithm that helps identify the most likely culprit drug based on criteria such as type of drug, timing and possible alternative causes (45, 46). An ALDEN score of 4 or more is usually required for the SJS/TEN phenotype.

#### Epidemiology

The incidence of SJS/TEN is estimated at 2–7 cases per million people per year using a German population based-registry with an increase prevalence of SJS cases compared to TEN (47, 48). Recently, data from the FDA adverse event reporting system (FAERS) indicated a rate of 0.15% with 30,202 reactions among the 20,406,852 adverse drug events reported in the database (49). In lower- and middle-income countries where TB and HIV are more prevalent, the rates of SJS/TEN are up to 10-fold higher (10).

#### Drugs

The agents most commonly implicated are allopurinol, anticonvulsants and antibiotics (50). However, in about one third of cases, a drug cannot clearly be associated with the development of the SJS/TEN (46).

#### Management

Following drug withdrawal and avoidance of cross-reactive medications, for SJS/TEN, given the multiorgan involvement, various specialties must be involved in the acute setting such as ophthalmology, head and neck, gastroenterology, gynecology, etc. Patients are usually transferred to burn units in order to be able to receive the adequate wound care, nutritional and fluid support (51, 52). The role of adjunctive therapies is unclear at this time with the use of systemic corticosteroids being controversial (47). While reports on mortality show contradictory results, a meta-analysis regrouping 1,209 patients indicated a benefit with corticosteroid treatment (decreased mortality) compared to supportive treatment alone (53). Intravenous immunoglobulins (IVIG), while part of the management in various centers, have an unclear clinical benefit (54). The combination of systemic corticosteroids and IVIG seems to be associated with the lowest mortality rates compared to each treatment alone (55). Cyclosporine has also been used with promising results in terms on mortality reduction (56, 57). Considering the high mortality rate for this condition, novel therapies are required. Recent studies have shown a possible benefit in the acute phase of the disease following the use of TNF-alpha inhibitors such as etanercept. These agents improved skin healing and decreased mortality as estimated by predictive scores (58).

### Generalized bullous fixed drug eruption

#### Clinical description

The generalized bullous fixed drug eruption (GBFDE) is considered a rare type of fixed drug eruption that is multifocal and widespread, characterized by sharply defined bullae at the same site following recurrent administration of offending drug (59). The skin surface under the large flaccid bullae is often widespread red or brown (59). Systemic symptoms such as fever and arthralgias have also been described. The main differential diagnosis for this condition is SJS/TEN but GBFDE has a milder course with rapid skin healing in absence of scarring following drug discontinuation (60, 61).

#### Epidemiology

While fixed drug eruption (FDE) has been commonly described with an incidence of 14–22% (61), the incidence of GBFDE is unknown at this time.

#### Drugs

Fixed drug eruption has been associated with numerous drugs from antibiotics to analgesics and NSAIDS as well as sedatives (61). In a cohort of 48 GBFDE cases, the mean time to disease after drug administration was 2.9 days and the suspected drugs varied from antibiotics to analgesics and NSAIDS (59).

#### Management

As for all the previously described conditions, the main treatment is culprit drug removal followed by symptomatic management to decrease pain or related pruritus (61). A biopsy excluding alternative cause (e.g., SJS/TEN, TEN-like lupus and immunobullous disease such as bullous pemphigoid, linear IgA disease) is required. The biological marker granulysin has been shown to help differentiate SJS/TEN from other conditions (62). While the aim of this review is to present diagnostic tools, the GBFDE has been presented as part of the differential diagnostic for SJS/TEN and will not discussed in detail in the subsequent sections.

## Diagnostic tools

### History and drug timeline

A detailed clinical history is crucial to diagnose drug-related reactions. For beta-lactam allergy, it has been demonstrated that beta-lactam allergy interviews, in absence of skin testing, can assist in ruling out an allergy and reduce the use of non-beta-lactam antibiotics such as fluoroquinolones, considered high-*Clostridioides difficile* infection-risk antibiotics (63, 64). However, this has been infrequently deployed in moderate to severe presumed T-cell mediated reactions.

Following a detailed history, assessing the temporal association between symptoms and drug exposure with the help of a drug timeline is crucial. Any drug started more than 6–8 weeks before the reaction is less likely to be causal (65). The drug half-life must also be considered. SJS/TEN reactions associated with drugs that have a long half-life (more than 20 h) have been associated with an increase in mortality (26%) compared to drugs with shorter half-life (5% mortality) (66). This suggests that the time of drug discontinuation is also important. Using validated causality scores such as the Naranjo score can help guide clinicians in identifying the culprit agents. All agents administered must be considered causal with recent reports showing that T-cell mediated reactions can rarely occur after the administration of agents such as proton pump inhibitors (67) or anti-histamine receptors such as ranitidine (68). Among the agents commonly used in the hospital setting, contrast agents are often reported to be culprit (69, 70). The nursing and the pharmacy team can provide valuable assistance with identifying the agents for the drug timeline. Further, the pharmacy team can assist with pharmacovigilance researches by exploring existing databases (71).

### *In vivo* allergy assessment tools

#### Intradermal testing

Previous prospective studies and both international and local allergy society guidelines support the use of skin prick and intradermal testing (IDT) for drug allergy assessment (72–80). The concentration administered is designed to cause the least amount of irritation as per published guidelines (72, 74, 81–83), although validated concentrations for T-cell mediated reactions are less well described. Further, the true concentrations required to induce a positive T-cell response are unknown with recent studies showing that the use drugs such as vancomycin at the highest non-irritating concentrations are not enough to evoke a T-cell mediated reaction at the injection site (84). All the agents used are usually approved by local health regulations and have been safely administered via the intradermal route (77, 85–89). However, the sensitivity and specificity of skin tests are not validated for non-immediate reactions and, apart from penicillin, there are no current standardized extracts for skin tests.

Intradermal testing implies that a small quantity (0.02–0.05 mL) of a drug at a non-irritant concentration is gently injected under the skin. The testing is usually performed on the volar surface of the forearm and it is recommended to keep sufficient space (approximately 2–2.5 cm) between each injected agent. The preferred area is 5 cm from the wrist and 3 cm from the antecubital fossa. An immediate reading is performed after 15–20 min and a delayed reading after 24–48 h. A positive reaction translates as erythema and a local reaction when compared with the injection of a negative control, usually saline. A histamine prick test is used as a positive control for immediate reactions and several medications such as antihistamines have been identified as being able to suppress this local reaction. In this context, all drug known to affect the skin testing should be stopped depending on the described duration of suppression. There is no positive control for delayed reactions.

For penicillin non-severe allergic reactions, performing testing with the major allergenic determinant (penicilloyl polylysine), a minor determinant mixture (penicillin G, penicilloate, penilloate), and amoxicillin translated to a negative predictive value of 97.9% (90) for immediate reactions. There is currently a clear recommendation for skin testing followed by challenge for pregnant women with a history of penicillin allergy considering the importance of a beta-lactam treatment for *Group B Streptococcus* (91–93). In a cohort of children with low risk beta-lactam delayed-type reactions, delayed IDT was considered a useful tool (94).

There is a clear role for delayed IDT reading in delayed reactions to penicillin with evidence showing that delayed reading would have identified an additional 25% of patients in a prospective cohort of 37 patients (95). Furthermore, there is increasing evidence that IDT is safe even for the severe delayed phenotypes (96, 97). Cases of disease reactivation with mild isolated skin symptoms following skin testing have been described, especially when the testing was performed in the first 4–6 weeks following the acute reaction (98). The sensitivity of delayed IDT for antimicrobials ranges from 40% (96, 99) to 56% (98) for the severe phenotypes, excluding SJS/TEN (Table 2). However, the specificity and the false positive rate are not known.

**TABLE 2:** Recent reported sensitivity and specificity for delayed intradermal testing in drug allergy.

Reference	Study	Patients	Conditions	Drug Category	Sensitivity	Specificity
Fransson et al. (95)	Prospective	57	MPE	Antibiotics	25%	n/a
Copaescu et al. (98)	Prospective	69	MPE AGEP DRESS GBFDE SJS	Antibiotics	46%	n/a
Trubiano et al. (157)	Prospective	32	MPE AGEP DRESS	Antibiotics	56%	n/a
Nakkam et al. (192)	Prospective	15	DRESS	Vancomycin	n/a	n/a
Konvinse et al. (84)	Retrospective	23	DRESS	Vancomycin	33%	n/a
Trubiano et al. (96)	Prospective	31	FDE AGEP DRESS SJS/TEN	Antibiotics	42%	n/a
Romano et al. (193)	Prospective	214	MPE AGEP Bullous exanthema TEN	Antibiotics	97%	n/a
Buonomo et al. (194)	Retrospective	97	MPE	Antibiotics	95%	n/a
Cabanas et al. (99)	Retrospective	3	DRESS	Antibiotics	100% (3/3)	n/a
Barbaud et al. (102)	Prospective	4	DRESS	Antibiotics	3/4	n/a

AGEP, acute generalized exanthematous pustulosis; DRESS, drug reaction with eosinophilia and systemic symptoms; GBFDE, generalized bullous fixed drug eruption; MPE, maculopapular exanthema; n/a, non-applicable; SJS, Stevens-Johnson syndrome; TEN, toxic epidermal necrolysis.

#### Patch testing

In patients considered sensitized or allergic, antigen specific T-cells can be found on the surface of the skin. By applying non-irritant drug allergen concentrations under occlusion on the intact skin, patch testing (PT) aims to reproduce in the small limited area of the test the original delayed reaction. The PT is usually applied on the back or lateral upper arm area. There is no positive control that has been used with PT but the testing uses a negative control such as petroleum gel. Patch testing is usually left in place for a duration of 48 h with some studies showing benefit of performing a 7-day reading especially for certain preservatives (100). This is a time-consuming process as patients are asked to avoid showers and an increase in heat/humidity.

Non-irritant concentrations of various drugs for use in patch testing have been established (101, 102). However, there are currently no international guidelines for PT preparation as to ensure the quality of the products with large differences in active ingredient concentrations when using commercially available pure drugs compared with commercialized forms (103). Some alternatives for the classic PT method have been provided such as the scratch-patch involving the scarification or stripping of the epidermis with specialized tapes prior applying the PT (104). While this method proved to be non-irritant compared to the PT, carefully consideration is required especially for the severe phenotypes such as SJS/TEN. Indeed, cases of disease reactivation following PT have been reported in the literature, particularly in the immunosuppressed population (105).

The current published clinical studies underline a low sensitivity of this tool while the specificity is elevated, favoring a role of this tool for the more severe immune-mediated hypersensitivity reactions (102, 106, 107) (Table 3). Another advantage of this tool, compared to the IDT, is the possibility to use non-sterile and oral drug formulations. It is also interesting to note that the positivity of this tool seems to depend on the assessed drug as well as the reported reaction (108). In the clinical setting, considering this low reported sensitivity, lack of a validated positive control and less than 100% negative predictive value, removal of the allergy label should not be performed following a negative PT. In the pediatric population, while the literature is very limited, the sensitivity seems to be lower compared to the adult population (94). A positive PT should help confirm an immunologic mechanism with studies showing an increased reproducibility with positive PT not been affected by the time interval between testing, sex or age (111, 112). However, this is still dependent on the drug and the use of patch testing, IDT and *ex vivo/in vitro* testing and genetic testing are likely to be complementary (109, 110).

**TABLE 3:** Recent reported sensitivity and specificity for patch testing in drug allergy.

Reference	Study	Patients	Conditions	Drug category	Sensitivity	Specificity
Gilisen et al. (107)	Retrospective	*N* = 9	MPE (6) AGEP (2) DRESS (1) *Healthy (78)	Clindamycin	100%	100%
Prasertvit et al. (195)	Retrospective	*N* = 20	HIV NVP hypersensitivity (20) *Healthy (15)	Nevirapine (NVP)	10%	100%
Ben Mahmoud et al. (192)	Retrospective	*N* = 20	MPE (11) DRESS (6) SJS (2) FDE (2) Erythroderma (2)	Antiepileptics	95%	n/a
Atanaskovic-Markovic et al. (94)	Prospective	*N* = 57 (pediatric)	MPE (57)	Antibiotics	32%	n/a
Hassoun-Kheir et al. (106)	Prospective	*N* = 25	MPE (13) SJS (4) DRESS (3) AGEP 1 FDE (2) Vasculitis (1) SDRIFE (1) *Healthy (25)	Antibiotics Antiepileptics	32%	92%
Buonome et al. (194)	Retrospective	*N* = 97	Delayed Reactions	Antibiotics	100%	n/a
Cabanas et al. (99)	Retrospective	*N* = 8	DRESS (8)	Piperacillin-Tazobactam	1/4 (25%)	n/a
Barbaud et al. (102)	Prospective	*N* = 134	DRESS (72) AGEP (45) SJS/TEN (17)	Antibiotics Corticosteroids Antiepileptics Other agents	57%	n/a

AGEP, acute generalized exanthematous pustulosis; DRESS, drug reaction with eosinophilia and systemic symptoms; FDE, fixed drug eruption; HIV, Human immunodeficiency virus; MPE, maculopapular exanthema; n/a, non-applicable; SDRIFE, Symmetrical drug-related intertriginous and flexural exanthema; SJS, Stevens-Johnson syndrome; TEN, toxic epidermal necrolysis. *Indicated the total number of patients.

#### Drug ingestion challenge test

Several protocols have been suggested for challenge testing in non-severe delayed reactions: (1) single step direct challenge (113–115), (2) 2-step graded challenge (116), (3) single or multiple step challenge following negative delayed intradermal skin testing/patch testing (117–120), (4) direct multiple days challenge or (5) multiple days challenge following negative skin testing (117, 121). In absence of an immediate objective reaction, the “immediate” protocols have often led to the removal of the allergy label even in the context on a reported delayed reaction.

The benefits of penicillin allergy assessment based on clinical history (in person or telemedicine visit) (122, 123), skin testing (124) and challenge have been demonstrated in various studies in recent years (122, 125). Furthermore, for the non-severe delayed reactions such as MPE, algorithms based on direct challenge (with no prior skin tests) are considered a safe and cost-effective option (64, 126–129). However, currently, there are no clear guidelines on the optimal assessment tools for these low risk penicillin allergies with a need to compare skin testing followed by oral challenge, if negative, to direct oral challenge. Pharmacist led protocols have been instrumental in providing safe and rapid in hospital delabeling (130–132). This literature has evolved from pediatric penicillin and aminopenicillin allergic cohorts, where direct challenge without skin testing is considered part of standard of care (133–135). In these non-severe cases, the presence of an underlying immune mechanism is unclear and the majority of the skin isolated drug eruptions could be related to a non-allergic condition such as a viral illness or a drug-viral interaction (13). For the pediatric population, there is a need to develop clinical decision scores that can be used outside the allergy clinic assessment as to allow improvement of antibiotic stewardship.

While the literature provides interesting evidence for the non-severe reactions, strict drug avoidance is still part of the recommendations for the severe phenotypes associated with an increased mortality (16, 65). In these cases, the use of structurally non-related drugs in recommended. In particular scenarios such as reported in a South African study with anti-tuberculosis drugs, drug re-challenge with empirically initiated intravenous corticosteroids following the first clinical signs has been associated with a majority of mild to moderate reactions (136, 137). There is also evidence that *ex vivo* assays such as the enzyme-linked immunoSpot (ELISpot) could help risk stratify patients providing diagnostic accuracy compared to the current gold standard, the drug ingestion challenge (138). Large, multi-center international studies are required to further characterize drug re-challenge as a tool to provide optimal drug treatment following *in vivo* and *ex vivo* testing.

### *Ex vivo* tools

#### Lymphocyte transformation test

The lymphocyte transformation test (LTT) has been widely used for past 30 years and is considered the forefather of *ex vivo* testing in drug allergy (139). It is reported that patient isolated memory T-cells can be stimulated with causal agents leading to a drug-specific T-cell proliferation. Because of this mechanism, the LTT is also addressed as a lymphocyte proliferation test of a lymphocyte stimulation or activation test (140). This cell proliferation is defined according to a stimulation index (SI) or the proportion between the drug stimulated lymphocytes and the background lymphocyte proliferation. This ratio aims to take into consideration the biological variation. For the classic LTT, it is calculated based on a radioactive uptake marker directly proportional to the degree of T-cell proliferation in response to a drug antigen (140). In recent years, variations of the LTT platform have been proposed in the literature.

The reported sensitivity of LTT in delayed hypersensitivity reactions ranges from 27% (141) to 74% (142) and specificity was quoted as 85–100% (141–144) (Table 4). When this tool was studied for a specific phenotype, its accuracy greatly improved. For example, in a cohort of 41 DRESS patients, the reported sensitivity was 73% and the specificity was 82%, using samples from a recovery phase and not an acute phase (145). Further, the sensitivity can vary depending on the drug studied and expression of either granulysin, granzyme B or IFN-γ. In a cohort of 63 patients with SCAR associated to the use of anti-epileptics, the sensitivity increased when using granulysin-based lymphocyte activation tests stimulated with carbamazepine (73.9%). Other experimental techniques to increase the sensitivity of this tool have been described such CTLA-4 blocking of lymphocytes, demonstrating the importance of T-cell regulatory pathways (146, 147).

**TABLE 4:** Reported cases and cohorts showing clinical advantage with the use of the LTT.

Author	*N*	Country	Phenotypes	Drug(s)	Controls	Sensitivity	Specificity
Cabanas et al. (145)	41	Spain	DRESS	Antibiotics Anticonvulsants Antifungals	n/a	73%	82%
Suthumchai et al. (155)	23	Thailand	DRESS (9) AGEP (4) SJS/TEN (10)	Allopurinol Anticonvulsants Antibiotics Other*	Non-allergic individuals (20)	52%	n/a
Ye et al. (196)	8	South Korea	MPE DRESS	Isoniazid or rifampicin	n/a	100%	n/a
Haw et al. (159)	16	UK	MPE (7) DRESS (5) SJS (3) SJS/TEN (1)	Antibiotics Anticonvulsants Antifungals	n/a	78%	n/a
Sun et al. (150)	57	China	MPE	Antituberculosis drugs	Control group (96)	23–58%	93–98%
Meller et al. (197)	22	Germany	MPE	Pegylated interferon	Control group (7)	23%	100%
Porebski et al. (144)	23	Poland	MPE	Anticonvulsants	Control group (24)	30%	100%
Cabanas et al. (99)	8	Spain	DRESS	Piperacillin-Tazobactam	n/a	100%	n/a
Porebski et al. (141)	15	Poland	SJS	Antibiotics Anticonvulsants	Control group (18)	27%	95%

Case reports were excluded from this table. AGEP, acute generalized exanthematous pustulosis; DRESS, drug reaction with eosinophilia and systemic symptoms; FDE, fixed drug eruption; MPE, maculopapular exanthema; n/a, non-applicable; SJS, Stevens-Johnson syndrome; TEN, toxic epidermal necrolysis. *Other drugs included tramadol, ibuprofen, and mefenamic acid.

The value of this test was exemplified in various cohort studies and case reports where this tool provided clinical assistance in determining the optimal drug options in both a pediatric (148, 149) and an adult population (141, 150). However, some of these cases can be subject to misclassification bias as the initial reported phenotype was not always consistent with a hypersensitivity reaction (148).

#### Enzyme-linked immunoSpot assay

The T-cell ELISpot assays measuring IFN-γ cytokine response to different agents has been used to assist drug hypersensitivity causality investigations in patients with drug allergy (143, 151–154). Compared to LTT, in an adult cohort of 23 SCAR patients, the ELISpot IFN-γ helped identify more drug-specific IFN-γ releasing cells (155). Similar to LTT, this laboratory technique requires viable well-preserved patient T lymphocytes and involves the use of complex manipulations for which an operator-dependent variability could influence the assay results.

In general, standardized concentrations for *ex vivo* diagnostics can be based on confirmatory data from performed cytotoxicity assays (97, 156). However, various studies using non-studied concentrations have been published. Given that antibiotics are a major culprit for SCAR, these agents have been commonly used for the ELISpot assays (98, 157, 158). Other commonly reviewed agents are anticonvulsants, antituberculosis drugs and allopurinol (141, 155, 158, 159).

Depending on the used definition and the studied drugs, the sensitivity of this assay varied from 35% (158) to 86% (84, 160) with a reported specificity of 100% (Table 5). As very few cohort studies from specialized centers are available, there is a need to further explore this promising *ex vivo* method. In the pediatric population, an interesting study regrouping a cohort of 9 SCAR and 7 MPE compared LTT with ELISpot in both an acute and post-recovery phase. The authors showed the ELISpot assay using IFN-γ and IL-4 as cytokine outputs, produced a higher drug-specific response contributing to the diagnosis of the culprit drugs (159). However, the sample size is relatively small and hence results are non-conclusive at this point.

**TABLE 5:** Reported cases and cohorts showing clinical advantage with the use of the ELISpot.

Author	*N*	Country	Phenotypes	Drug(s)	Controls	Sensitivity	Specificity	Conclusions
Copaescu et al. (98)	63	Australia	MPE (17) AGEP (5) DRESS (34) SJS (5) TEN (1) GBFDE (1)	Antibiotics	Tolerant controls (5)	54%	100%	IFN-γ positive in 34/63 (≥50 SFU/10^6^)
Trubiano et al. (157)	12	Australia	DRESS (3) AGEP (3) MPE (6) B-lactams	Antibiotics	n/a	42%	n/a	IFN-γ positive in 5/12 (≥50 SFU/10^6^)
Klaewsongkram et al. (158)	116	Thailand	DRESS (50) AGEP (16) SJS/TEN (50)	Antibiotics Allopurinol Antituberculosis drugs Anticonvulsants	Non-allergic control drugs from 62 SCAR patients	35%	n/a	IFN-γ positive in 19/50 DRESS, 4/16 AGEP and 18/50 SJS/TEN (>95% CI controls SFCs)
Konvinse et al. (84)	23	United States	DRESS (14)	Vancomycin	n/a	86%	n/a	IFN-γ positive in 12/14 (≥50 SFU/10^6^)
Suthumchai et al. (155)	23	Thailand	AGEP (4) DRESS (9) SJS/TEN (10)	Allopurinol Anticonvulsants Antibiotics Other*	Non-allergic control (20)	70%	n/a	IFN-γ positive in 17/23 (>18 SFU/10^6^)
Trubiano et al. (97)	19	Australia	AGEP (2) DRESS (14) SJS (2) TEN (1)	Antibiotics	Tolerant controls (16)	52%	100%	IFN-γ positive in 10/19 patients and 0/16 controls (>50 SFU/10^6^)
Xiong et al. (198)	1	China	SJS (1)	Sulphapyridine	n/a	n/a	n/a	IFN-γ positive (300 spots/10^6^)
Trubiano et al. (199)	1	Australia	TEN (1)	Teicoplanin	n/a	n/a	n/a	IFN-γ positive (≥50 SFU/10^6^)
Ye et al. (196)	8	South Korea	MPE (4) DRESS (4)	Antituberculosis drugs	n/a	63%	n/a	IFN-γ and GrbB positive (>0 Spots/10^4^ cells) for T-cell clones with reactivity for INH/RFP (5/8)
Kato et al. (200)	16	Japan	MPE (1) DRESS (5) EM-like (7) SJS/TEN (3)	Allopurinol Antibiotics Anticonvulsants Celecoxib	n/a	19%	100%	IFN-γ positive in 3/16 patients
Haw et al. (159)	16	UK	MPE (7) DRESS (5) SJS/TEN (4)	Antibiotics Antifungals Anticonvulsants	n/a	IFN-γ: 77% IL-4: 85%	n/a	IFN-γ positive in 14/18 patients IL-4 positive in 11/13 patients
Klaewsongkram et al. (201)	24	Thailand	DRESS (13) SJS/TEN (11)	Allopurinol	Controls (21)	71%	95%	IFN-γ positive in 15/24 (>16 SFU/10^6^) and 1/21 controls
Porebski et al. (144)	23	Poland	MPE (23)	Anticonvulsants	Tolerant controls (24)	GrB: 55% Grl: 39.1%	100%	GrB positive in 12/22 (SFU > 50) Grl positive in 9/22
Lucas et al. (202)	12	Australia	Abacavir HSR HLA-B*57:01 Positive	Abacavir	HLA-B*57:01 + Abacavir naive (3), HLA B*57: 01 tolerant (15) or Abacavir naïve (9)	100%	97–100%	IFN-γ positive in 12/12 (>10 SFU10^6^) and 0/3, 1/15 and 0/9 controls
Ben-Said et al. (13)	21	France	DRESS (9) MPE (12)	Antibiotics	n/a	71%	n/a	IFN-γ positive in 9/9 DRESS and 6/12 MPE
Keane et al. (203)	19	Australia	Nevirapine HSR	Nevirapine	n/a	40%	n/a	Nevirapine-specific responses were detected in 4/12 (>100 SFU/10^6^)
Tanvarasethee et al. (204)	25	Thailand	MPE (15)	Cephalosporins	Non-allergic controls (20)	24% (IFN-γ or IL-5) 40% (IFN-γ and IL-5)	100%	IFN-γ and IL-5 positive in 10/25 (mean > 20 SFU/10^6^)
Porebski et al. (141)	15	Switzerland	SJS/TEN (15)	Allopurinol (1) Anticonvulsants (9) Sulfonamide (4) Mefenamic acid (1)	Drug-exposed controls (18)	GrB: 33% Grl:40% (NKp46+) Grl: 53% (CD3+CD4+)	95–100%	GrB positive in 5/15 patients Grl positive in 6/15 (NKp46+) patients and 8/15 (CD3+CD4+)
Phatharacharukul et al (205)	1	Thailand	DRESS (1)	Sulfasalazine	n/a	n/a	n/a	IFN-γ positive (1 048 SFU/10^6^)
El-Ghaiesh et al. (160)	8	UK	Piperacillin HRS (8)	Piperacillin	Tolerant controls (5)	87–100%	100%	IFN-γ positive in 8/8 (>10 SFU/10^6^); 7/8 (>30 SFU/10^6^)
Bensaid et al. (152)	1	France	DRESS (1)	Amikacin	n/a	n/a	n/a	IFN-γ positive (213 SFU/10^6^)

AGEP, acute generalized exanthematous pustulosis; DRESS, drug reaction with eosinophilia and systemic symptoms; ELISpot, enzyme linked ImmunoSpot; EM, erythema multiforme; GBFDE, generalized bullous fixed drug eruption; GrB: Granzyme B; Grl, granulysin; HSR, hypersensitivity; MPE, maculopapular exanthema; n/a, non-applicable; SFU, spot forming unit; SJS, Stevens-Johnson syndrome; TEN, toxic epidermal necrolysis. *Other drugs are tramadol, ibuprofen (2) and mefenamic acid.

The increase in serum level of the IFN-γ cytokine in conditions such as MPE and SJS/TEN has been previously documented (161). But other cytokines have been identified such as IL-8, IL-17, and IL-22 in AGEP (162–164), IL-4, IL-5, IL-13, and TARC In DRESS (165, 166) and IL-15 in SJS/TEN (167, 168). This provides relevance for possible outputs to explore in functional assays as to increase the sensitivity of these tools.

#### Genetic testing

There have been an increasing number of HLA associations described with many drugs and SCAR (Table 6). Some examples include HLA-B*57:01 screening prior prescription of the anti-retroviral drug abacavir (169–171) and HLA-B*15:02 screening before carbamazepine prescription in many South-East Asian countries where this allele is prevalent (172, 173). A study from Thailand reported that 21.2% of SCAR could have been prevented by screening for HLA-B alleles prior to drug exposure (158). Recently, studies have reported that DNA methylation, identified using genome-scale methylation analysis, might play a role in allopurinol SJS/TEN (174). The presence of HLA-B*58:01 is considered a predisposing factor for developing allopurinol/oxypurinol induced SCAR in Southeast Asian populations but not in European and African ancestry populations (175).

**TABLE 6:** Human leukocyte antigen (HLA) associations in delayed drug hypersensitivity.

Author (year)	Drug	HLA	Phenotype	Ethnicity	Screening	NPV (%)	PPV (%)	NNT
Mallal et al. (206)	Abacavir	B*57:01	AB HS	Caucasian (5–8%) African/Asia (<1%) African American (2.5%)	**Routine screening** HIV Positive patients	100	55	13
Chung et al. (62)	Carbamazepine	B*15:02^c^	SJS/TEN	Han Chinese (10–15%) Koreans, Japanese (<1%) European Ancestry (<0.1%)	**Routine screening** in Southeast Asian countries	100	3	1000
Hung et al. (190)	Allopurinol	B*58:01	SJS/TEN DRESS	Han Chinese (9–11%) European ancestry (1–6%)	Selective screening^b^	100	3	250
Zhang (191)	Dapsone	B*13:01	DRESS	Papuans/Australian aborigines (28%) Chinese (2–20%) Japanese (1.5%) Indian (1–12%) African and African American (<2%)	**Routine screening** for leprosy patients in countries with increased prevalence	99.8	7.8	84
Konvinse et al. (84)	Vancomycin	A*32:01	DRESS	European ancestry (6.8%) African American (4%) Southeast Asian (<1.5%)	Pre-emptive^a^	99.99	0.51	75

AB HS, abacavir hypersensitivity syndrome; HIV, human immunodeficiency virus; NNT, numbers needed to test (to prevent one case); NPV, negative predictive value; PPV, positive predictive value. ^a^HLA-A*32:01 testing could have a role in determining the culprit drug (vancomycin) when multiple drugs are implicated in a delayed hypersensitivity reaction. ^b^The American College of Rheumatology has recommended preventive screening for patients of Korean ethnicity with chronic kidney disease stage 3 or worse and patients of Han Chinese or Thai ethnicity irrespective of renal function before starting allopurinol (207). ^c^Other described alleles: HLA-B*15:21, HLA-B*15:11, and HLA-B*15:18.

Vancomycin induced DRESS was associated with the expression of HLA-A*32:01 (84) and evidence shows that vancomycin directly interacts with naïve T-cells expressing HLA-A*32:01 (176). In the Thai population, HLA-B*15:02, HLA-C*06:02, HLA-C*08:01, and HLA-B*13:01 were associated with co-trimoxazole hypersensitivity reactions and mostly SJS/TEN (177, 178). Dapsone and its reactive metabolite, nitroso dapsone, induced hypersensitivities such as DRESS in individuals with HLA-B*13:01 (179, 180). Carbamazepine triggered SCAR, was linked to HLA-A*31:01 in Caucasian and Japanese populations (181). Following genome-wide association studies, HLA-B*57:01 and HLA-B*57:03 were reported in patients with drug-induced liver injury caused by flucloxacillin (182, 183) and (HLA)-DRB1*01:01 has been associated with nevirapine-induced hepatic hypersensitivity reactions (184). Anti-osteoporotic agents induced SJS were suggested to be associated with HLA-A*33:03 (185).

However, genetic screening is not currently integrated in routine practice and a comprehensive description of the current identified genetic markers is beyond the scope of this review. The biggest concern with HLA screening for many drugs is the fact that HLA risk is necessary but not sufficient for the development of the hypersensitivity in question. In many cases this means that an extremely high number of patients would need to be tested in order to prevent one case of hypersensitivity and hence this is not a cost-effective confirmatory test. However, there could be scenarios where HLA testing could be used beyond screening and could have a diagnosis role such as the HLA-A*32:01 testing for vancomycin DRESS in the setting of multiple implicated drugs.

### Lessons learned from *in vivo* and *ex vivo* drug diagnostic tools

**FIGURE 3:**
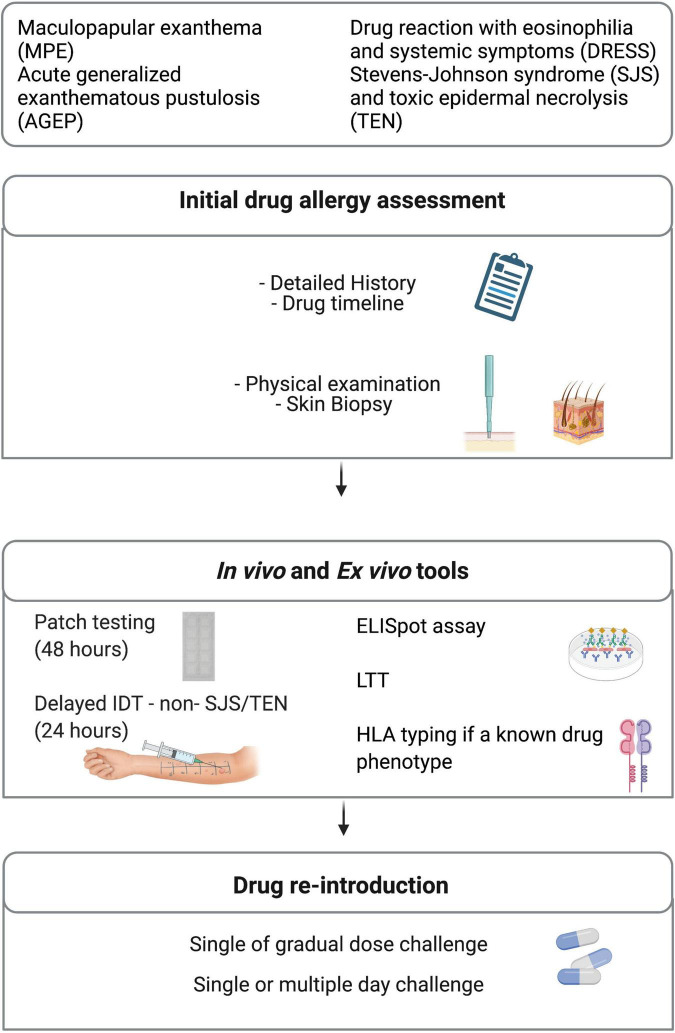
Diagnostic management. ELISpot, enzyme-linked immunoSpot; HLA, human leukocyte antigen; IDT, intradermal testing; LTT, Lymphocyte transformation test.

Drug allergy labels have important impact on patient care by limiting not only the use of appropriate medications but also by increasing costs and quality of patient care (10, 124, 186). A multidisciplinary patient-centered risk/benefit-based assessment must be part of the management plan (Figure 3). What is the optimal management for the patient’s acute condition? What is the reported reaction or described phenotype and what was the most likely causal drug? If the culprit drug is stopped, are there any other drug alternatives available for the patient? Another important inquiry often unexplored is regarding the patient’s willingness to take the medication or alternative drugs again. Unfortunately, the clinical investigations can sometimes be limited by the patient’s refusal of *in vivo* investigations. In this scenario, *ex vivo* tools are appealing as the safety of the procedure can be guaranteed (Figure 3). However, as discussed, these tools are not available in the majority of health facilities. Another limit of these tools is their lack of validity. It is possible that the low sensitivity of these diagnostic tools is due to the fact that current assays rely on drug or drug metabolites that are not effectively recognized by the immune system (187). Also, considering that none of these diagnostic tools have a 100% negative predictive value, their use should aim to complement each other as to improve the sensitivity and the specificity of the diagnosis.

There is a current need to provide internationally accepted management algorithms for *in vivo* and *ex vivo* diagnostic tools and/or challenge while understanding the possibility that these algorithms might not apply to all phenotypes. The currently available tools must be prospectively used as to allow safe drug re-introduction.

## Conclusion

Despite the increased mortality associated with SCAR, diagnostic tools remain limited and unstandardized. Ongoing research is required to better understand the epidemiology, the diagnostic approach and management strategies for these delayed drug reactions. Furthermore, large scale studies validating clinical diagnostic tools used for DHR are required.

## Author contributions

AC performed the literature review and wrote the manuscript text with supervision from MB-S and JT. All authors reviewed the manuscript, made a substantial, direct, and intellectual contribution to the work, and approved the manuscript for publication.

## Abbreviations

AGEP: acute generalized exanthematous pustulosis
DRESS: drug reaction with eosinophilia and systemic symptoms
ELISpot: enzyme-linked ImmunoSpot
MPE: maculopapular exanthema
SCAR: severe cutaneous adverse reaction
SJS: Stevens-Johnson syndrome
TEN: toxic epidermal necrolysis.
